# Supplementary material for: Diagnostic performance of the AID line probe assay in the detection of Mycobacterium tuberculosis and drug resistance in Romanian patients with presumed TB
Source: PLoS One. 2022 Aug 10;17(8):e0271297. doi: 10.1371/journal.pone.0271297 (PMC9365181; doi:10.1371/journal.pone.0271297)
Supplement: S3 File — (DOCX) [file pone.0271297.s003.docx]

**Online supplement**

***Methods***

***Inclusion & exclusion criteria***

Inclusion criteria

- Having chest radiograph compatible with pulmonary TB plus one of the following
- Having signs and symptoms indicative for TB: productive cough for more than two weeks, haemoptysis, fever, night sweats or substantial involuntary weight loss.
- Being willing to be tested for HIV-infection
- Provided written informed consent to study participation.

Exclusion criteria were

- Being on active anti-TB treatment (consisting of at least two active compounds) in the past 6 months
- Suffering from physical or mental inability to attend study visits (as determined at the discretion of the investigator)
- Being a member of a vulnerable population group (prisoner, soldier, under guardianship, < 18 years)
- Having extrapulmonary TB without pulmonary involvement
- Being incapable of producing two sputum samples of sufficient quality (mucoid) and volume (at least 3 ml).

***Microbiological methods for M. tuberculosis detection***

All sputum samples were examined by microscopy for the presence of AFBs using auramine-rhodamine fluorochrome staining. AFB-positive results were confirmed by Ziehl-Neelsen staining (Henry D. Isenberg LSG. Clinical Microbiology Procedures Handbook. American Society for Microbiology. 2007;1) and scored using WHO/IUATLD guidelines. For evaluation of mycobacterial growth in culture, all collected samples were inoculated on solid Lowenstein Jensen (LJ) medium (manufactured by the National Institute of Research & Development for Microbiology & Immunology “Cantacuzino” (NIRDMI) EUHEM, Bucharest) and in liquid culture VersaTREK^TM^ Myco bottles containing growth (VersaTREK Myco GS) and antibiotic supplements (VersaTREK Myco PVNA) (TREK Diagnostic Systems, Cleveland, OH, USA).

***Reader rules AID assay***

AID assay reader rules ensuring consistent results interpretation were established. For an *M. tuberculosis* positive result, the conjugate control and the *M. tuberculosis* complex signal needed to be present. In the case of a negative *M. tuberculosis* test result, the amplification control band needed to be present next to the conjugate control band. The mycobacterium universal band was not considered in the interpretation of AID-test results. In the case of *M. tuberculosis* detection, the reader rules allowed to analyse each drug resistance independently. This means, if the wildtype or mutated hybridization result for all tested genetic sequences of a specific drug (e.g. RIF) were available, the result was considered valid even if the respective bands for another drug of the same module (e.g. INH) did not give a valid signal.

***Data analysis***

Case definitions:

Patients in the microbiological confirmed TB group had at least one *M. tuberculosis* positive culture result (either on LJ or Versatrec) for at least one out of two analyzed sputum samples. Patients in the “No TB” category showed no growth of *M. tuberculosis* in any sputum sample and experienced clinical recovery without anti-TB treatment and/or received an alternative medical diagnosis explaining their respiratory symptoms. In patients with clinical TB there was no microbiological proof of *M. tuberculosis*, however, anti-TB treatment was initiated based on clinical grounds. Patients that did not fit into any of the other categories were regarded as indeterminate.

**Supplement table 1: Patterns of indeterminate AID test results**

| **Sub-groups (n)** | **Non-evaluable tests AID INH/RIF (n)** | | **Non-evaluable tests AID FQ/EMB (n)** | | **Non-evaluable tests AID AG (n)** | |
| --- | --- | --- | --- | --- | --- | --- |
|  | M.tb detection  (n=139) | Resistance detection in AID M.tb-positives (n=46)** | M.tb detection  (n=139) | Resistance detection in AID M.tb-positives (n=46)*** | M.tb Detection  (n=139) | Resistance detection in AID M.tb-positives (n=46)**** |
| M.tb negative (69) | 3 | - | 0 | - | 6 | - |
| M.tb positive (59)* | 0 | 13 RIF-resistance  13 INH-resistance | 0 | 28 EMB-resistance  36 FQ-resistance | 1 | 9 STR-resistance  5 KAN/AMK- resistance  3 CAP-resistance |
| - Smear-pos. (43)* | 0 | 11 RIF-resistance  11 INH-resistance | 0 | 27 EMB-resistance  31 FQ-resistance | 1 | 4 STR-resistance  2 KAN/AMK- resistance  1 CAP-resistance |
| - Smear-neg. (16) | 0 | 2 RIF-resistance  2 INH-resistance | 0 | 1 EMB-resistance  5 FQ-resistance | 0 | 5 STR-resistance  3 CAN/AMK- resistance  2 CAP-resistance |
| Clinical TB (10) | 0 | - | 0 | - | 1 | - |
| **Participants (n (%)) with non-evaluable test result** | **3 (2.19%)** | **15 (32.61%)** | **0 (0.0%)** | **36 (78.26%)** | **8 (5.76%)** | **9 (19.57%)** |

**Legend to supplement table 1: ***58 culture confirmed M.tb. positives (42 of them being smear-positive) were included in the evaluation of the AG module; ******15 participants had any non-evaluable resistance results (either for RIF or INH) by the INH/RIF-module, in 11 participants the resistance testing for both drugs (INH and RIF) was non-evaluable (on the same strip); ***28 participants had non-evaluable results for both drugs (EMB and FQ) on the same strip, while 8 participants had only a non-evaluable test for the detection of FQ-resistance testing by the EMB/FQ-module; ****three participants had non-evaluable resistance results for STR, CAP and KAN/AMK by the AG-module (on the same strip), 2 participants had a non-evaluable result for KAN/AMK and STR on the same strip and 4 participants had a non-evaluable result for STR resistance only.
